# Sinonasal Disease in Pediatric Patients With Cystic Fibrosis and the Cystic Fibrosis Transmembrane Conductance Regulator (CFTR) I1234V Mutation in Qatar

**DOI:** 10.7759/cureus.88481

**Published:** 2025-07-21

**Authors:** Mohammad Odeh, Amal R Al-Naimi, Abbas Younes

**Affiliations:** 1 Pediatric Pulmonology Medicine, Sidra Medicine, Doha, QAT; 2 Pediatric Pulmonary Medicine, Sidra Medicine, Doha, QAT; 3 Otolaryngology, Sidra Medicine, Doha, QAT

**Keywords:** cftr mutation, cystic fibrosis (cf), ear nose throat (ent), pediatric pulmonology, sino nasal

## Abstract

Background and objective

Sinonasal disease is a common but underrecognized manifestation in patients with cystic fibrosis (CF). The CF transmembrane conductance regulator (CFTR) I1234V mutation is the most prevalent CF mutation in Qatar, yet its specific impact on upper airway pathology remains poorly characterized. This study aimed to evaluate the prevalence and clinical features of sinonasal disease in pediatric patients with CF who are homozygous for the CFTR I1234V mutation in Qatar.

Methodology

A retrospective cross-sectional study was conducted at Sidra Medicine, including pediatric patients diagnosed with homozygous CFTR I1234V mutation between September 1, 2017, and June 30, 2024. Clinical data, sinonasal symptoms, ENT examination findings, computed tomography (CT) imaging results, and surgical interventions were collected and analyzed.

Results

A total of 29 patients were included (16, 55.1%, female, mean age 10.4 years). More than half (16, 55.1%) were asymptomatic for sinonasal complaints. However, 13 (44.8%) reported nasal congestion, and 8 (27.5%) had nasal polyps on endoscopy. CT scans performed in nine patients revealed sinusitis in all cases (100%) and polyposis in seven (77.7%). Surgical intervention was required in 8 (27.5%) patients, primarily functional endoscopic sinus surgery. Co-colonization with methicillin-resistant Staphylococcus aureus (MRSA) and Pseudomonas was noted in three (10.3%) patients.

Conclusions

Pediatric patients with CF who are homozygous for the I1234V mutation in Qatar exhibit variable expression of sinonasal disease, with many demonstrating radiologic or endoscopic abnormalities despite being asymptomatic. These findings highlight the importance of routine ENT assessment in this population, even in the absence of complaints, to guide early intervention and potentially reduce respiratory complications.

## Introduction

Cystic fibrosis (CF) is a life-threatening autosomal recessive disorder that primarily impacts the respiratory and digestive systems. It results from mutations in the CF transmembrane conductance regulator (CFTR) gene, impairing the transport of chloride and sodium across the cell membrane. This dysfunction causes the accumulation of thick, sticky mucus, leading to persistent respiratory infections and inflammation, especially in the airways and sinuses [1].

Sinonasal complications, particularly chronic rhinosinusitis (CRS) and nasal polyps, are commonly seen in patients with CF. CRS involves long-standing inflammation of the nasal and sinus mucosa, often persisting beyond 12 weeks despite treatment, and is associated with symptoms such as nasal congestion, facial discomfort, nasal discharge, and impaired sense of smell [2]. Nasal polyps, benign mucosal growths, frequently develop in patients with CF and can further worsen airway obstruction and sinus-related symptoms [3].

In Qatar, the CFTR I1234V mutation stands out as the most prevalent CF-related genetic variant [3], making it a unique and significant area of research. However, its specific influence on sinonasal pathology remains inadequately characterized. Investigating this mutation's impact may unveil distinct clinical patterns and contribute to developing mutation-specific diagnostic and therapeutic approaches, a focus that sets this study apart.

Although global research has addressed sinonasal involvement in CF, few studies have explored its relationship to specific CFTR mutations. Sinonasal manifestations may substantially impair patients’ quality of life, contributing to sleep disorders, cognitive issues, and increased disease burden [1]. Additionally, viscous mucus promotes bacterial colonization, perpetuating recurrent infections and complicating disease control.

Computed tomography (CT) remains a cornerstone in evaluating sinonasal disease in CF. It provides detailed visualization of sinus opacification, mucosal changes, and polyp formation, guiding medical management and surgical planning [3].

This study contributes to understanding the specific effects of the CFTR I1234V mutation on sinus disease in pediatric patients with CF in Qatar. By examining the prevalence and severity of rhinosinusitis and nasal polyps and analyzing CT imaging findings, we have gained valuable insights into the clinical implications of this mutation. These findings can directly inform and enhance care strategies for affected children, underscoring the practical significance of this research.

## Materials and methods

This retrospective cross-sectional study was conducted at Sidra Medicine, a tertiary pediatric hospital in Qatar. We reviewed the records of all pediatric patients diagnosed with CF who were homozygous for the CFTR I1234V mutation and followed in the CF clinic between September 1, 2017, and June 30, 2024. Inclusion criteria were: (1) a confirmed diagnosis of cystic fibrosis based on clinical features and sweat chloride testing; (2) genetic confirmation of homozygosity for the CFTR I1234V mutation; (3) age 18 years or younger at the time of data collection; and (4) regular follow-up at Sidra Medicine during the study period.

Exclusion criteria included patients with CFTR mutations other than I1234V, patients older than 18 years, and those with incomplete or missing clinical or genetic data. Clinical and demographic data were extracted from the hospital’s electronic medical record (EMR) system. Extracted demographic variables included age, sex, and body mass index (BMI) *z*-scores. Clinical data were obtained from pulmonology documentation and included lung function, radiologic findings, microbiological results, and treatment history. Lung function data included forced expiratory volume in one second (FEV₁), measured only in patients aged six years and older, by institutional guidelines. FEV₁ values were expressed as percent predicted.

CF was defined based on clinical features consistent with CF and confirmed by sweat chloride concentration ≥60 mmol/L on two separate occasions and/or identification of two disease-causing CFTR mutations, as per international guidelines. In our study, we only included patients with CFTR I1234V homozygous mutation. Recurrent hospital admission was defined as two or more hospital admissions for CF-related respiratory symptoms within 12 months. Microbiological data were collected from the electronic medical record and included results from sputum and bronchoalveolar lavage (BAL) cultures. Identification of pathogens, including Pseudomonas aeruginosa, Staphylococcus aureus, methicillin-resistant Staphylococcus aureus (MRSA), and non-tuberculous mycobacteria (NTM). ENT-related data were collected from otolaryngology consultation or clinic visit notes. They included reported symptoms (e.g., nasal congestion, mouth breathing, snoring, epistaxis) and physical examination findings (e.g., nasal polyps, septal deviation, adenoidal or tonsillar hypertrophy). Sinus CT results and records of surgical interventions (e.g., adenoidectomy, functional endoscopic sinus surgery) were also reviewed. Laboratory investigations included liver function tests, serum immunoglobulin E (IgE) levels, and 25-hydroxy vitamin D levels.

Statistical analysis

Descriptive statistical analysis was performed using Microsoft Excel. Continuous variables were reported as mean values with minimum and maximum ranges. Categorical variables were presented as frequencies and percentages. Patients with incomplete key variables were excluded from the corresponding analyses. No imputation was performed for missing data.

Ethical considerations

The study was approved by the Institutional Review Board of Sidra Medicine (IRB #2231075). The requirement for informed consent was waived due to the retrospective nature of the study and minimal risk to participants.

## Results

We identified 29 patients (16 females and 13 males) with confirmed diagnosis of CF with homozygous CFTR I1234V mutation. The mean (range) age at the time of the study was 10.4 (1.17-18.4) years. The mean (range) BMI *z*-score was -0.11 (-2.5 to 2.4). Pancreatic insufficiency was diagnosed in four patients (13.7%). Additionally, recurrent hospital admissions occurred in seven patients (24.1%), and abnormal liver function tests were observed in 10 patients (34.4%). Microbiological findings revealed positive MRSA nasal screening in 14 patients (48%), and 10 patients (34.4%) were also positive for MRSA on sputum culture. On sputum culture, Pseudomonas aeruginosa was isolated in seven patients (24.2%), and three (10.3%) had co-infection with both MRSA and Pseudomonas. BAL culture was performed in 17 patients: 6 (35.3%) were positive for Staphylococcus aureus, 2 (11.7%) for MRSA, 3 (17.6%) for Pseudomonas aeruginosa, and 2 (11.7%) had a combination of MRSA and Pseudomonas aeruginosa. Non-tuberculous mycobacteria (NTM) were isolated in three patients, all of whom grew Mycobacterium abscessus; one patient also had a co-infection with Mycobacterium avium. All three NTM-positive cases had positive sputum cultures, and two also tested positive for Mycobacterium abscessus on BAL culture. On chest CT imaging, bronchiectasis was observed in 16 patients (57.1%), while nodular opacity and bronchial wall thickening were found in one (3.5%) and three patients (10.7%), respectively. Eight patients (28.6%) had normal chest CT findings. Detailed demographic and clinical characteristics are summarized in Table 1.

**Table 1:** Clinical characteristics of the study participants (n = 29). BMI, body mass index; IgE, immunoglobulin E; CFRD, cystic fibrosis-related diabetes; FEV₁, forced expiratory volume in 1 second; MRSA, methicillin-resistant Staphylococcus aureus; NTM, nontuberculous mycobacteria; BAL, bronchoalveolar lavage; CT, computed tomography

Characteristics	
Age per years at time of study, mean (range)	10.4 (1.17-18.4)
Male, *n* (%)	13 (44.8%)
Female, *n* (%)	16 (55.1%)
BMI *z*-score, mean (range)	-0.11 (-2.5 to 2.4)
Sweat chloride mean (range)	82.6 (61-107)
Pancreatic insufficiency, *n* (%)	4 (13.7%)
CFRD, *n* (%)	4 (13.7%)
Recurrent admission, *n* (%)	7 (24.1%)
FEV_1_ % of predicted, mean (range)	82% (42%-103%)
IgE level, mean (range)	168 (2-1782)
Vitamin D level, mean (range)	62.5 (24.4-134)
Abnormal liver function tests, *n* (%)	10 (34.4%)
Positive MRSA screening from the nares, *n* (%)	14 (48%)
Positive NTM from sputum culture, *n* (%)	3 (10.3%)
Positive MRSA from sputum culture, *n* (%)	10 (34.4%)
Positive Pseudomonas aeruginosa from sputum culture, *n* (%)	7 (24.1%)
Combination Pseudomonas aeruginosa and MRSA, *n* (%)	3 (10.3%)
BAL culture, *n* (%)	17 (58.6%)
Combination of positive culture for both MRSA and pseudomonas, *n* (% among patients who had BAL culture)	2 (11.7%)
MRSA, *n* (% among patients who had BAL culture)	2 (11.7%)
Pseudomonas aeruginosa, *n* (% among patients who had BAL culture)	3 (17.6%)
Staphylococcus aureus, *n* (% among patients who had BAL culture)	6 (35.3%)
Chest CT scan, *n* (%)	28 (96.5%)
Bronchiectasis, *n* (% among patients who had CT chest)	16 (57.1%)
Bronchiectasis nodular opacity, *n* (% among patients who had CT chest)	1 (3.5%)
Bronchial wall thickening, *n* (% among patients who had CT chest)	3 (10.7%)
Normal result, *n* (% among patients who had CT chest)	8 (28.5%)

More than half of patients denied sinonasal symptoms (16 patients, 55.1%). The rest had multiple varying symptoms, most commonly nasal congestion, including 13 patients (44.8%), mouth breathing in three patients (10.3%), snoring in seven patients (24.1%), and epistaxis in two patients (6.8%). On the sinonasal exam, septal deviation with tonsillar enlargement was found in three patients (10.3%), and in one patient, isolated tonsillar enlargement was found (3.4%). Diagnostic nasal endoscopy detected nasal polyps in eight patients (27.5%) and a submucosal abscess in one patient (3.4%). Sinus CT scans were performed on nine patients (31%), all of whom showed evidence of sinusitis (9/9 patients, 100%). Among them, seven patients (77.7%) had nasal polyposis. The most common surgical intervention was functional endoscopic sinus surgery in seven patients (24.1%). Three had adenotonsillectomy (10.3%), and four had polypectomy (13.7%). One patient (3.4%) needed isolated grommet insertion. It is important to note that some patients underwent more than one surgical intervention. Post-adenotonsillectomy persistent symptoms were reported in 2 out of 3 patients (66%). ENT (ear, nose, and throat) clinical findings, imaging results, and surgical interventions are summarized in Table 2.

**Table 2:** ENT clinical findings, imaging, and surgical intervention among study patients (n = 29). CT, computed tomography; ENT, ear, nose, and throat

ENT characteristics	
Asymptomatic with normal exam	16 (55.1%)
Congested nose	13 (44.8%)
Mouth breathing, *n* (%)	3 (10.3%)
Snoring, *n* (%)	7 (24.1%)
Epistaxis, *n* (%)	2 (6.8%)
Apnea, *n* (%)	0 (0%)
Septal deviation, *n* (%)	3 (10.3%)
Tonsils enlargement, *n* (%)	4 (13.7%)
Ear discharge, *n* (%)	2 (6.8%)
Polyposis on endoscopy, *n* (%)	8 (27.5%)
Submucosal abscess on endoscopy, *n* (%)	1 (3.4%)
Surgical intervention, *n* (%)	8 (27.5%)
Functional sinuses surgery, *n* (% among patients who had surgical intervention)	7 (24.1%)
Adenotonsillectomy, *n* (%)	3 (10.3%)
Grommet insertion, *n* (%)	1 (3.4%)
CT sinuses, *n* (%)	9 (31%)
Sinusitis, *n* (% among patients who had CT sinuses)	9 (100%)
Polyposis, n (% among patients who had CT sinuses)	7 (77.7%)

## Discussion

Studies of clinical phenotype in CF in correlation with CFTR genotype have revealed a very complex relationship [1]. The I1234V mutation is a missense mutation in exon 19, replacing adenine with guanine at nucleotide 3832 of the CFTR gene and replacing the isoleucine residue at codon 1234 with a valine residue [1]. This mutation causes a processing and trafficking defect by activating a cryptic donor splicing site that leads to a truncated exon 19 [4]. Altered CFTR splicing leads to alternative transcripts lacking 18 nucleotides, which are translated into a protein lacking 6 amino acids [4]. This mutation is particularly significant in Qatar and parts of the Middle East, where it is among the most common CFTR variants, especially in homozygous form [4]. Unlike the more severe F508del mutation, I1234V is often associated with a relatively milder pulmonary phenotype and preserved pancreatic function, although variability exists [5,6].

Sinonasal disease is a well-recognized yet underreported manifestation of CF. It includes CRS, nasal polyposis, and recurrent sinus infections [7]. These complications arise from defective mucociliary clearance and persistent inflammation, creating an environment favorable for microbial colonization [2]. Previous research suggests that CFTR mutations, especially classes I and II, are linked to more severe sinonasal symptoms [8]. However, there is little data on the specific sinonasal impact of the I1234V mutation.

In our cohort of 29 pediatric patients with CF who were homozygous for the CFTR I1234V mutation, more than half (55.1%) were asymptomatic concerning sinonasal complaints, suggesting that this mutation may be associated with a milder clinical expression of upper airway disease. Nevertheless, 13 (44.8%) patients experienced nasal congestion, and 8 (27.5%) had nasal polyps confirmed by diagnostic nasal endoscopy. The percentage of nasal polyps was double what previous studies showed for milder mutations [8] and approached two-thirds of the rate observed in the general CF population, where Weber and Ferrari [9] reported a prevalence of 39%.

Notably, all patients who underwent sinus CT (9, 31% of the cohort) had radiological evidence of sinusitis, which is much higher than the general population (10.9%) [10]. Seven patients (77.7%) also had nasal polyps. This finding supports the theory that even without overt symptoms, radiologic and endoscopic evidence of sinonasal disease may be present, which aligns with earlier studies, suggesting subclinical sinus involvement in CF [9].

Surgical intervention was required in approximately one-quarter of our patients. Functional endoscopic sinus surgery (FESS) was the most common procedure, performed in seven (24.1%) patients, often accompanied by adenoidectomy, tonsillectomy, and/or polypectomy. This rate is comparable to or slightly lower than surgical rates reported in cohorts with more severe mutations [11], suggesting a possibly milder sinonasal disease course in I1234V patients. However, 66% of patients who underwent adenotonsillectomy complained of persistent symptoms postoperatively, raising questions about the long-term efficacy of specific surgical approaches in this population. This may indicate the need for better preoperative selection criteria or more aggressive postoperative management strategies.

An interesting aspect of our data was the overlap between sinonasal disease and microbial colonization. Nearly half of the cohort (14, 48%) had positive MRSA screening from nasal swabs, and seven (24.2%) had Pseudomonas aeruginosa colonization. The presence of both pathogens in three (10.3%) patients is particularly concerning, given the role these organisms play in accelerating lung function decline and increasing morbidity in CF [12]. Whether upper airway colonization contributes directly to lower airway infection remains unclear, but studies have suggested potential seeding from the sinuses to the lungs [13]. Thus, aggressive management of sinonasal disease might also aid in reducing pulmonary exacerbations.

This study adds to the limited literature on the sinonasal manifestations of the I1234V mutation in pediatric patients with CF, especially in the Middle East. Using clinical, endoscopic, and radiologic assessments allows for a more comprehensive evaluation of sinonasal disease. However, our study has limitations, including a relatively small sample size and retrospective design. Not all patients underwent sinus CT or nasal endoscopy, which may have led to an underestimation of true disease prevalence. Additionally, there is no direct comparator group with other CFTR mutations, limiting the ability to draw definitive genotype-specific conclusions.

## Conclusions

Pediatric patients with CF who are homozygous for the CFTR I1234V mutation in Qatar display a heterogeneous pattern of sinonasal disease. While over half are asymptomatic, many have endoscopic or radiologic evidence of CRS and nasal polyposis. This highlights the importance of routine ENT surveillance in all patients with CF, regardless of symptom burden. Surgical intervention remains a key treatment in selected cases, but recurrence and persistent symptoms postoperatively necessitate ongoing follow-up and individualized care plans. Future research should focus on prospective, multicenter studies comparing the I1234V mutation to other CFTR mutations and examining whether early sinonasal intervention can mitigate pulmonary complications and improve quality of life. Additionally, studies should investigate the role and efficacy of CFTR modulators in treating patients with this specific mutation.

## References

[1] Ratjen F, Doring G (2003). Cystic fibrosis. Lancet.

[2] Elborn JS (2016). Cystic fibrosis. Lancet.

[3] Fokkens WJ, Lund VJ, Mullol J (2012). EPOS 2012: European position paper on rhinosinusitis and nasal polyps 2012. A summary for otorhinolaryngologists. Rhinology.

[4] Hammoudeh S, Gadelhak W, AbdulWahab A, Al-Langawi M, Janahi IA (2022). An overview of the homozygous cystic fibrosis transmembrane conductance regulator mutation c.3700 A>G (p.Ile1234Val) in Qatar. Manara - Qatar Research Repository.

[5] Abdul Wahab A, Al Thani G, Dawod ST, Kambouris M, Al Hamed M (2001). Heterogeneity of the cystic fibrosis phenotype in a large kindred family in Qatar with cystic fibrosis mutation (I1234V). J Trop Pediatr.

[6] El Bar Aluma B, Sarouk I, Senderowitz H (2020). Phenotypic and molecular characteristics of CF patients carrying the I1234V mutation. Respir Med.

[7] Krajewska J, Zub K, Słowikowski A, Zatoński T (2022). Chronic rhinosinusitis in cystic fibrosis: a review of therapeutic options. Eur Arch Otorhinolaryngol.

[8] Berkhout MC, van Rooden CJ, Rijntjes E, Fokkens WJ, el Bouazzaoui LH, Heijerman HG (2014). Sinonasal manifestations of cystic fibrosis: a correlation between genotype and phenotype?. J Cyst Fibros.

[9] Weber SA, Ferrari GF (2008). Incidence and evolution of nasal polyps in children and adolescents with cystic fibrosis. Braz J Otorhinolaryngol.

[10] Hastan D, Fokkens WJ, Bachert C (2011). Chronic rhinosinusitis in Europe--an underestimated disease. A GA²LEN study. Allergy.

[11] Tipirneni KE, Woodworth BA (2017). Medical and surgical advancements in the management of cystic fibrosis chronic rhinosinusitis. Curr Otorhinolaryngol Rep.

[12] Ji KS, Frank-Ito D, Abi Hachem R (2022). Endoscopic sinus surgery for cystic fibrosis: variables influencing sinonasal and pulmonary outcomes. Am J Rhinol Allergy.

[13] Mainz JG, Hentschel J, Schien C, Cramer N, Pfister W, Beck JF, Tümmler B (2012). Sinonasal persistence of Pseudomonas aeruginosa after lung transplantation. J Cyst Fibros.

